# On Tenia and Teniafuges

**Published:** 1881-07

**Authors:** Carl Bettleheim


### Selections.

### ON TENIA AND TENIAFUGES.

BY DR. CARL BETTELHEIM.

THE determination of the species—whether Tenia solium or Tenia mediocanellata, is unnecessary, and I have found that the expulsion of one variety is no more difficult than that of the other.

Having satisfied yourself of the existence of a tapeworm in your patient, the next point is to consider any symptoms which may contra-indicate the treatment for its expulsion.

Contra-indications are: any anatomical lesion of the stomach (gastric ulcer, acute catarrh, etc.); all febrile affections, wet-nursing, convalescence from severe illness, the menstrual period in females, pregnancy, weakness from old age, and in sickly children, if the presence of the worm does not produce serious inconvenience. Anæmia, if not too extreme, is not a positive contra-indication.

If there are no symptoms which would lead you to delay an attempt at expulsion, you should then appoint a time for the same, when you may be at leisure to give it your entire attention, because if you are unable to do so, you may be disappointed in the results; something may occur which will cause a failure, you must remember that your remedies only sicken the worm for a time, and that if it recovers before expulsion is effected, it may attach itself to the walls of the intestines, and thus frustrate all the efforts which you have made. Carefully examine all fecal discharges. I have known attendants to declare that they contained no worm, when upon an examination I have found them to contain the entire worm with the head all rolled into a small mass.

My cure lasts from forty minutes to somewhat over four hours. I order the patient to abstain from all food (water excepted) for from eighteen to twenty-four hours before beginning the anthelmintic medication, and at the same time give suitable purgatives, the best of which are, in my estimation, castor oil or powd. jalap; a tablespoonful of the oil or from 0.3 to 0.5 (4½ to 8 grains) of the powder, morning, noon and night, and another dose three or four hours before giving the anthelmintic. The latter I prescribe as follows:

Cort. rad. punic granat., 2—400=(℥vj–xij). Macerate for twenty-four hours in aquæ destill., 5—600=(f.℥xvj–xix), and then evaporate until the strained residue measures 200—300 =(f.℥vj–ix). Care should be taken that the bark used is fresh, as it deteriorates by age.

The resulting decoction, which should be of a clear, dark-brown color, is administered by means of an œsophageal tube, which is introduced to about the middle of the œsophagus and the fluid poured into the same by means of a small glass funnel. The patient is then directed to remain as quiet as possible, and if the remedy is not vomited within half an hour or an hour, the worm is generally expelled in from three-quarters to four and a quarter hours.

If the bowels do not move in an hour and a half after the administration of the medicine, another dose of the purgative or an enema should be given; this should, however, not be done sooner, as it may cause vomiting.

Grown persons generally allow the œsophageal tube to be introduced without much remonstrance, but some, of course, have an uncontrollable dread of such a procedure, and will rather drink the entire dose than submit to it.

If the decoction is vomited within a short time after its introduction into the stomach, 3 or 4 grams (45 to 60 grs.) of ethereal extract of male fern, in pill form, or 60 to 75 grs. of knissin, in powder, should be given in 0.5 gram (7½ grs.) doses every half hour to hour. The success of these remedies is, however, not as rapid nor as sure as my method.

Should the worm be only partially expelled and hang from the anus, the administration of anthelmintics must be continued. The patient should sit upon two chairs, and the worm carefully rolled on to a stick; as soon as the worm resists this procedure, having fastened itself to the intestinal walls by means of its suckers and hooks, the patient should be directed to lie upon his left side, and an enema of lukewarm water, to which a few drops of turpentine have been added, should be given, forcing the same as far up the intestinal canal as possible.

If the worm should not be expelled because the medicine is vomited as soon as taken, the same procedure should be repeated in a few days, only then the anthelmintic must be introduced into the stomach in three or four portions, at intervals of fifteen or thirty minutes.

For children I use a smaller quantity of the decoction, 100 to 150 grams (3 to 5 fluid ounces), and if vomiting is produced too early, I continue it in doses of 20 to 30 grams (5 to 8 drachms), every forty to ninety minutes.

As soon as the worm is expelled, the patient should be allowed to partake of some good nourishing soup.

